# Supplementary material for: The interactome of intact mitochondria by cross-linking mass spectrometry provides evidence for coexisting respiratory supercomplexes
Source: Mol Cell Proteomics. 2017 Dec 8;17(2):216–32. doi: 10.1074/mcp.RA117.000470 (PMC5795388; doi:10.1074/mcp.RA117.000470)
Supplement: Supplemental Data [file supp_RA117.000470_133922_0_supp_23978_fzffwf.zip › spectra_annotation/mito_DR_spectra_annotation/265-1-8-1-5-1.pdf]

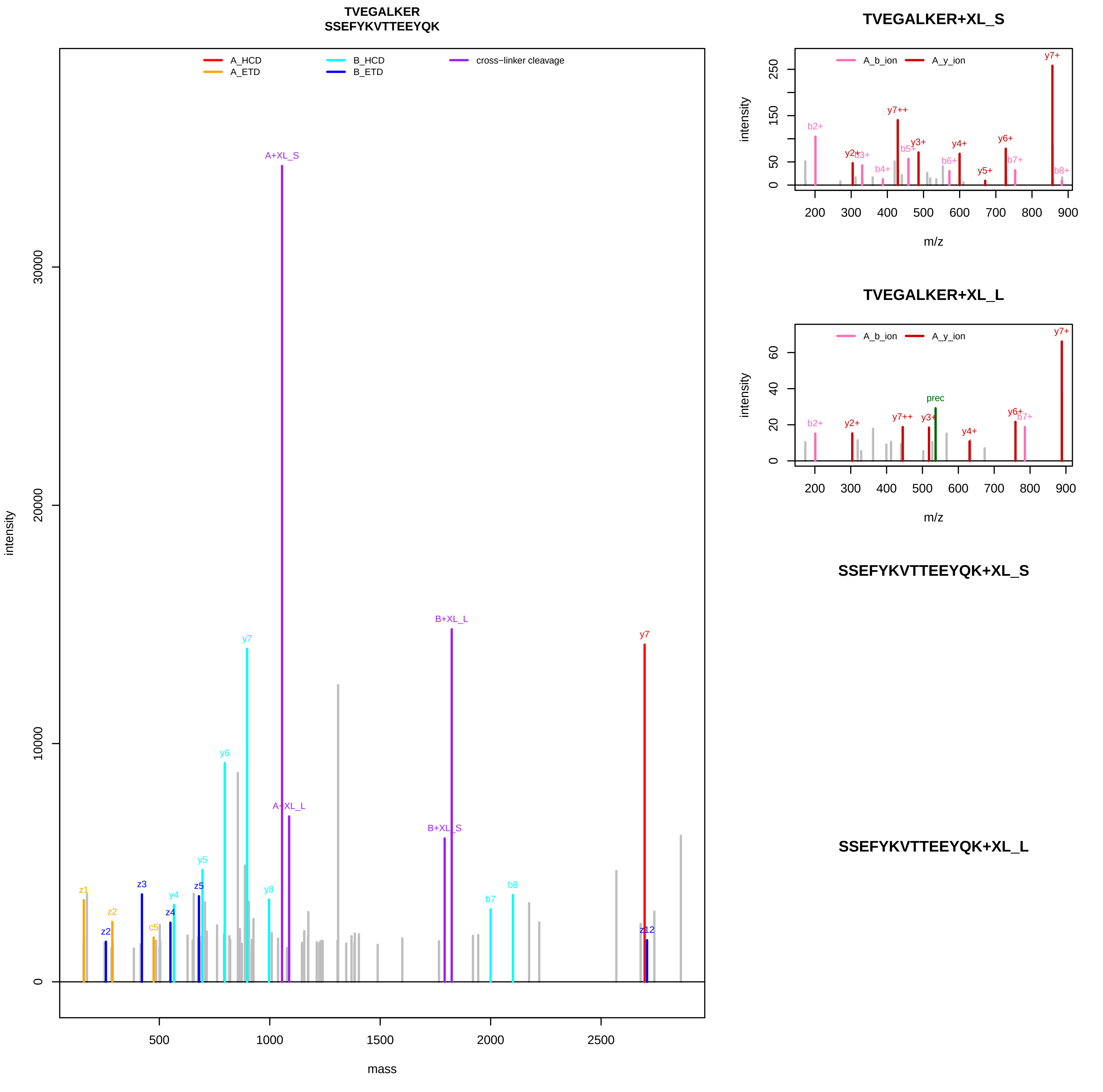
TVEGALKER
SSEFYKVTTEEYQK
A_HCD
A_ETD
B_HCD
B_ETD
cross−linker cleavage
intensity
mass
A+XL_S
A+XL_L
B+XL_L
B+XL_S
y7
y6
y5
y4
y8
b7
b8
z1
z2
z3
z4
z5
z12
c5
TVEGALKER+XL_S
A_b_ion
A_y_ion
m/z
b2+
y2+
b3+
b4+
y7++
b5+
y3+
b6+
y4+
y5+
y6+
b7+
y7+
b8+
TVEGALKER+XL_L
prec
SSEFYKVTTEEYQK+XL_S
SSEFYKVTTEEYQK+XL_L
